# Effects of progesterone on hyperoxia‐induced damage in mouse C8‐D1A astrocytes

**DOI:** 10.1002/brb3.435

**Published:** 2016-02-01

**Authors:** Friederike Weber, Stefanie Endesfelder, Christoph Bührer, Monika Berns

**Affiliations:** ^1^ Department of NeonatologyCharité University Medical CenterBerlinGermany

**Keywords:** Astrocytes, cell proliferation, hyperoxia, progesterone, progesterone receptor

## Abstract

**Introduction:**

The birth of most mammals features a dramatic increase in oxygen while placenta‐derived hormones such as *β*‐estradiol and progesterone plummet. In experimental newborn animals, transiently elevated oxygen concentrations cause death of neurons, astrocytes, and oligodendrocyte precursors. High oxygen has been associated with cerebral palsy in human preterm infants while progesterone is being used to prevent preterm delivery and investigated as a neuroprotective agent.

**Methods:**

In this study, we investigated the effects of hyperoxia (80% O_2_ for 24, 48, and 72 h) on cultured C8‐D1A astrocytes in the presence or absence of progesterone at concentrations ranging from 10^−9^ to 10^−5 ^mol/L.

**Results:**

Hyperoxia measured by methytetrazolium assay (MTT) reduced cell viability, increased release of lactate dehydrogenase (LDH), reduced carboxyfluorescein diacetate succinimidyl ester (CFSE)‐assessed cell proliferation, and downregulated Cylin D2 expression. Progesterone did not affect any of these hyperoxia‐mediated indicators of cell death or malfunctioning. Real‐time PCR analysis showed that hyperoxia caused downregulation of the progesterone receptors PR‐AB und PR‐B.

**Conclusions:**

Our experiments showed that there was no protective effect of progesterone on hyperoxia‐inducted cell damage on mouse C8‐D1A astrocytes. Down regulation of the progesterone receptors might be linked to the lack of protective effects.

## Introduction

Neurodevelopmental impairment is the major long‐term morbidity of preterm infants, with the risk for special educational needs rising strongly with decreasing gestational age (MacKay et al. 2010). For a long time, much of this problem had been attributed to intraventricular hemorrhage and cystic periventricular leukomalacia. However, of preterm infants below 1000 g birth weight but a normal head ultrasound, nearly 30% have been found to display cerebral palsy or low mental skills (Laptook et al. 2005). Thus, physiological changes resulting from leaving the intrauterine environment that strike a preterm infant prematurely are being investigated as noxious stimuli for the developing brain.

The placenta supplies the fetus with high amounts of 17*β*‐estradiol and progesterone that increase during the course of the gestation. After delivery of a premature infant, this placental supply is disrupted, resulting in a rapid decrease in both 17*β*‐estradiol and progesterone. Replacement of these placental hormones aiming at intrauterine conditions has been hypothesized to be beneficial. Supplementation of 17*β*‐estradiol and progesterone has been suggested to help improve neurocognitive outcome in preterm infants who are prematurely deprived of maternal estrogen. In extremely low birth weight infants who had been randomized to receive or not to receive postnatal replacement of 17*β*‐estradiol and progesterone, neurodevelopmental follow‐up at 5 years corrected age suggested a significant time–response relationship between hormone replacement and cerebral palsy, with every day of treatment significantly reducing the risk for cerebral palsy (Trotter et al. 2012).

The birth of mammals is associated with a rapid increase in oxygen availability, featuring a sustained 3–4 fold rise of oxygen tension within a few minutes. For the preterm infant, this normoxia is already a hyperoxic environment as the fetus is only exposed to a comparatively low oxygen concentration. The developing brain of the preterm infant is therefore exposed to increased oxygen levels weeks or months before the postnatal oxygen surge would be anticipated. We have repeatedly shown in both cultured cells and newborn rodents that an oxygen increase in similar magnitude causes cell damage to the immature rat brain, killing neurons, astrocytes, and oligodendrocyte precursors (Felderhoff‐Mueser et al. 2004; Gerstner et al. 2006; Huppmann et al. 2008; Schmitz et al. 2011; Yousuf et al. 2013). 17*β*‐estradiol ameliorates this oxygen toxicity in the infant rat brain. This protective effect is reversed by the estradiol antagonist tamoxifen and cannot be reproduced by 17*α*‐estradiol (Asimiadou et al. 2005). 17*β*‐estradiol produces significant dose‐dependent protection against oxygen‐induced apoptotic cell death of primary oligodendrocytes and attenuates loss of myelin basic protein observed with high oxygen (Gerstner et al. 2007). Protective effects of 17*β*‐estradiol for the neonatal brain have been also been observed after other insults, such as hypoxia–ischemia (Nunez et al. 2007). In contrast, the effects of progesterone are less well characterized. Progesterone has been reported to be neuroprotective after focal or global hypoxia/ischemia (Jiang et al. 1996; Gonzalez‐Vidal et al. 1998; Gibson and Murphy 2004; Ishrat et al. 2009), experimental trauma (Thomas et al. 1999), poisoning (Ishihara et al. 2012), and infections (Yousuf et al. 2013). Progesterone, however, has also been reported to block estrogen‐mediated protection (Carroll et al. 2008), possibly via downregulating estrogen receptors (Jayaraman and Pike 2009). Thus, it is important to dissect the action of progesterone on individual cell lineages.

In the set of experiments reported here, we first investigated the effect of transient hyperoxia on cultured murine C8‐D1A astrocytes, hypothesizing that increased oxygen is apt to cause apoptosis and impede proliferation. We then asked if hyperoxia‐mediated cell damaged could be rescued or ameliorated by progesterone.

## Materials and Methods

### Cell culture and treatment

The C8‐D1A astrocyte cell line (astrocytic type I clone, GFAP positive) was cloned from mouse cerebellum and purchased from American Type Culture Collection (ATCC CRL‐2541, LGC Standards, Wesel, Germany) (Alliot and Pessac 1984). Cells were cultured in Dulbecco's modified Eagle medium (DMEM; Biochrom, Berlin, Germany) containing glucose, 10% heat‐inactivated calf serum (FCS; Biochrom) and 2% L‐glutamine (Biochrom). To exclude interference of any steroid hormones possibly present in FCS, FCS was charcoal filtered prior to use (Activated charcoal C 9157, dextran D 1390; Sigma‐Aldrich, St. Louis, MO) (Eckert and Katzenellenbogen 1982). Phenol red‐free medium (RPMI; Biochrom) was used in all experiment in hyperoxia as well as in experiment with progesterone.

Cells were maintained as monolayers at 37°C, 5% CO_2_/15% N_2_/21% O_2_ (normoxia) or 5% CO_2_/15% N_2_/80% O_2_ (hyperoxia) with medium replacement every 2–3 days.

Water‐soluble progesterone (P 7556; Sigma‐Aldrich) was dissolved as a stock solution of 1 mmol/L in distilled aqua and diluted to concentrations of 10^−5^ mol/L, 10^−7^ mol/L, and 10^−9^ mol/L in culture medium before use. RU486 (Sigma‐Aldrich) was prepared in dimethylsulfoxide (DMSO, Sigma Aldrich) as a stock solution of 10 mmol/L, which was diluted to the working concentration of 10 *μ*mol/L with culture medium prior to use.

### Experiment set‐up

Astrocytes were seeded into 96‐wells microtiter plates (30,000/well in 100 *μ*L medium) and incubated for 24 h in normoxia. Progesterone or RU486 was added in the mentioned concentrations. The plates were incubated for further 24–72 h. To measure lactate dehydrogenase (LDH) release, 50 *μ*L of the cell culture supernatant was collected from each well and transferred to a fresh plate. The remaining 50 *μ*L of each well were used for colorimetric viability assays.

### Analysis of cell viability

#### Colorimetric viability assay

After exposure of astrocytes in 96‐well plates to hyperoxia or normoxia, measurements of reduction in 3‐(4,5‐dimethylthiazol‐2‐yl)‐2,5‐diphenyl tetrazolium bromide (MTT; Sigma‐Aldrich) were performed to estimate cell viability. MTT is reduced by active mitochondria in vital cells and consequently, the amount of formazan is proportional to the number of living cells. MTT was prepared as a 5 mg/mL stock solution in phosphate‐buffered saline (PBS; Biochrom). A quantity of 50 *μ*L of each well was collected and transferred to a corresponding microtiter plate for LDH release assay.

A quantity of 5 *μ*L of MTT was added to each well (final concentration 0.5 mg/mL) for 3 h. The reaction was stopped by 50 *μ*L 10% Sodium Dodecyl Sulfate (SDS; Sigma‐Aldrich). Absorbance was measured after incubation in a microplate reader (Bio‐Rad, Munich, Germany) at 570 nm, with a reference wavelength of 630 nm.

### Analysis of cell death

#### Lactate dehydrogenase (LDH) release

The lactate dehydrogenase release was measured using cytotoxicity detection kit (Roche Diagnostics, Mannheim, Germany). The test is based on the measurement of LDH activity released from damaged cells. Triton‐X 100 served as positive control. LDH release was calculated as percent of total release with Triton‐X 100. Absorbance was detected in a microplate reader (Bio‐Rad) at 490 nm and a reference wavelength 600 nm.

#### Cell death detection ELISA^plus^

To verify the results of the LDH release assay and to differentiate necrosis and apoptosis, we performed the sandwich‐enzyme‐immunoassay ELISA for cell death detection. Apoptosis was detected in the cell lysate, necrosis in the supernatant of the cell culture medium of each well. The test uses mouse monoclonal antibodies directed against DNA and histones (Anti‐histone‐biotin and Anti‐DNA‐POD – peroxidase‐conjugated monoclonal antibody from mouse), which allow the specific determination of mono‐ and oligonucleosomes in the cytoplasmatic fraction of cell lysates. Mono‐ and oligonucleosomes are released into the cytoplasm of cells that die from apoptosis, whereas necrotic cells do not show nucleosomal fragmentation (Salgame et al. 1997; Heinrich et al. 2009). The antibodies bind to the components building an immunocomplex binding to the streptavidin‐coated plate. ELISA assay was performed as described in the guidelines (Roche, Cell Death Detection ELISA^plus^ Manual). Absorbance was measured with a microplate reader at 405 nm and 490 nm.

### Analysis of cell proliferation

#### Flow cytometry

For analysis of cell proliferation, C8‐D1A astrocytes were labeled with carboxyfluorescein diacetate succinimidyl ester (CFSE; Molecular Probes, Eugene, Oregon), which is passed down to daughter cells during cell division. CFSE diffuses into astrocytes where acetate groups are split by intracellular esterases. The result is the fluorescent carboxyfluorescein succinimidyl ester which loses intensity of fluorescence with increasing cell proliferation (Lyons and Parish 1994). To identify maximum fluorescence of nonproliferated cells, fresh astrocytes were labeled with CFSE as described prior to measurement.

A 5 mmol/L stock solution of CFSE, diluted in DMSO (Sigma Aldrich) was prepared. Prior to use, the working concentration of 5 *μ*mol/L was diluted in PBS. Confluent astrocytes were harvested, centrifuged (5 min, 1000 rpm), and resuspended in 5 mL of prewarmed (37°C) CFSE solution with a concentration of 10^−6^ cells/mL. Cells were incubated for 15 min at 37°C, loading solution was replaced with medium and 200,000 astrocytes were seeded in each well of a 6‐well plate for further investigation. After exposure to 21% O_2_ or 80% O_2_ (24–48 h), cells were mechanically removed from the microtiter plate, centrifuged, resuspended in 1 mL PBS, and transferred to a BD Falcon 5 mL round‐bottom tube. We performed flow cytometry with 10,000 cells per measurement using FACS Canto II flow cytometer (Becton Dickinson Biosciences, San Jose, CA).

### RT‐PCR

Astrocytes were cultured in RPMI medium with 10% charcoal‐filtered FCS and 2% L‐Glutamin until cells were confluent. Cellular RNA was isolated using InviTrap Spin Universal MiniKit (Stratec molecular, Berlin, Germany) and DNA was removed with DNAse I (Roche). Using the One‐Step RT‐PCR Kit (Qiagen), the resulting cDNA (1 *μ*L) was amplified with polymerase chain reaction (PCR) using the oligonucleotide primers for progesterone receptor PR‐AB and PR‐B (TIB MOLBIOL, Berlin, Germany) shown in Table 1 (PR‐AB^#^ and PR‐B^#^). PCR‐mediated cDNA amplification was done in 40 cycles (each 60 sec) comprising 94°C for denaturing, 60°C for annealing, and elongation at 72°C. Amplified cDNA was applied to agarose gel electrophoresis (1% (w/v) agarose gel containing 0.00003% (v/v) ethidium bromide (Roth, Karlsruhe, Germany), which was also used for fluorescence measuring and qualitative analysis. PCR products were visualized and photographed using the UV recorder UV solo and Video Copy Processor.

**Table 1:** Primer

Gene	Oligonucleotide sequences 5′–3′	Gene bank database
PR‐AB^b^		
Forward	CAg Tgg Tgg ATT TCA TCC ATg	NM_008829.2 a
Reverse	CTT CCA gAG ggT Agg Tg	
PR‐B^b^		
Forward	ggA ggC AgA AAT TCC AgA CC	NM_008829.2 a
Reverse	gAC AAC AAC CCT TTg gTA gC	
PR‐AB^c^		
Forward	TCC gCT TCT AAA gAg CAA ACCT	NM_008829.2 a
Reverse	Agg AgC AgA AAA CCg TgA ATCT	
Probe	FAM TgT CAC TCT ggT CCC AAA TAMRA	
PR‐B^c^		
Forward	CTC ATA ggg AAg gAg gCA gAAA	NM_008829.2 a
Reverse	ggA ggg AgT CAA CAA CgA gTC TAA	
Probe	FAM AgC AgA CTC TTA gAC AgT gT TAMRA	
Cyclin D2		
Forward	CgT ACA TgC gCA ggA TggT	NM_009829.3
Reverse	AAT TCA Tgg CCA gAg gAA AgAC	
Probe	FAM Tgg ATg CTA gAg gTC TgT gA TAMRA	
mHPRT		
Forward	ATC ATT ATg CCg Agg ATT Tgg AA	NM_013556.2
Reverse	TTg AgC ACA CAg Agg gCCA	
Probe	FAM‐Tgg ACA ggA CTg AAA gAC TTg	
	CTC gAg ATg TAMRA	
*β*‐Actin		
Forward	CCC TAA ggC CAA CCg TgA AAA AgA Tg	NM_007393.3
Reverse	gAA CCg CTC gTT gCC AAT AgT GA Tg	

a In order to elucidate which progesterone isoforms is predominantly expressed in the C8‐1DA astrocyte cell lines, we amplified two isoforms of the PR‐AB and PR‐B.

b Oligonucleotide sequence used for RT‐PCR in normoxia (Fig. 3A).

c Oligonucleotide sequence used for quantitative RT‐PCR in hyperoxia (Fig. 3B).

### Quantitative real‐time PCR

For quantitative measurement of progesterone receptors, astrocytes were cultivated in 6‐well plates and exposed to either 21% or 80% O_2_ for 24 h. Total astrocytic RNA was extracted from snap‐frozen probes using phenol–chloroform extraction. To reduce contamination with DNA, probes were DNAse I treated (Roche). Transcription of mRNA into cDNA took place for 1 h at 42°C in the presence of 1 *μ*L M‐MLV reverse transcriptase (200 U/*μ*L) and 0.5 *μ*L RNAsin (40 U/*μ*L) (Promega, Madison, WI).

Real‐time PCR was carried out using KAPA^™^ PROBE FAST Universal qPCR Mastermix (Kapa Biosystems, Woburn, MA). Samples were labeled with 6‐carboxy‐fluoresceine (FAM) at 5′0 end and the quencher 6‐carboxy‐tetramethylrhodamine (TAMRA) at 3′ end. Probes were investigated as triplicates in a 96‐well plate. The following cycles were performed: 3 min at 95°C for denaturation and activation of polymerases, 45 cycles á 15 sec at 95°C for cyclic denaturation, and 45 cycles á 1 min at 65°C for hybridization and extension of oligonucleotides. The quantification of mRNA‐expression of PR‐AB, PR‐B, Cycline D2, and the housekeeping gene hypoxanthine‐guanine phosphoribosyltransferase (HPRT) was carried out using standard 2^−ΔΔCT^ method (Livak and Schmittgen 2001). Internal control was carried out by *β*‐actin control‐PCR. The primers are listed in Table 1 and were obtained from BioTez (Berlin, Germany).

### Statistical analysis

Data were analyzed in GraphPad Prism 6.05 (GraphPad Software, San Diego, CA, RRID:SCR_002798). The diagrams shown are the summaries of all essays. Experiments were repeated as indicated. Values are presented as mean and standard error of the mean (SEM). We performed Student's *t*‐test or one‐way ANOVA test with Bonferroni post hoc test to compare significant differences between groups. *P* < 0.05 was considered statistically significant.

## Results

### Effects of hyperoxia on C8‐D1A astrocytes

#### C8‐D1A astrocytes lose viability after exposure to hyperoxia (80% O_2_)

**Figure 1:**
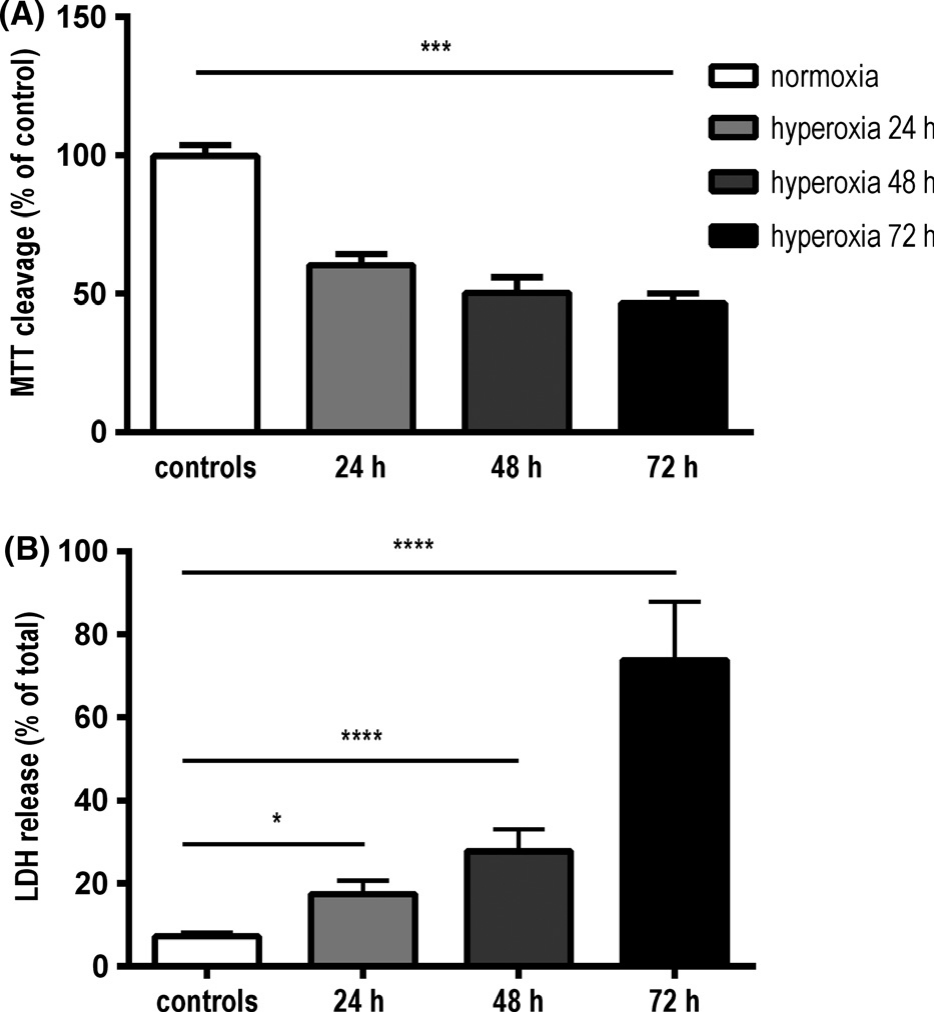
(A) Colorimetric viability assay (metabolism of MTT) after 24, 48 and 72 h in hyperoxia. C8‐D1A astrocytes showed a time‐dependent significant loss of cell viability compared to control cells in normoxia. (data are means ± SEM;* n* = 5; one‐way ANOVA with Bonferroni post hoc test; ***P < 0.001). (B) Cytotoxicity detection test (LDH release) after 24, 48 and 72 h in hyperoxia. Astrocytes showed a time‐dependent significant increase in LDH release. (data are means ± SEM;* n* = 5; one‐way ANOVA test with Bonferroni post hoc test; *P < 0.05, ****P < 0.0001).

Measurements of methyltetrazolium absorption revealed a significant loss of viability after exposure to hyperoxia for 24 h compared to controls (60.3 ± 4.1%; *P* < 0.001). The decline of vital astrocytes showed a time‐dependent progression. Incubation for 48 and 72 h in hyperoxia reduced cell viability further (50.3 ± 5.8%; 46.5 ± 3.7%; *P* < 0.001) (Fig. 1A). Cell death, measured by LDH release, increased significantly after 24, 48, and 72 h of exposure compared to control cells (17.4 ± 0.8%; *P* < 0.05; 27.7 ± 1.6%, and 73.8 ± 4.0%; *P* < 0.0001) (Fig. 1B).

#### Hyperoxia induces apoptosis in C8‐D1A astrocytes

**Figure 2:**
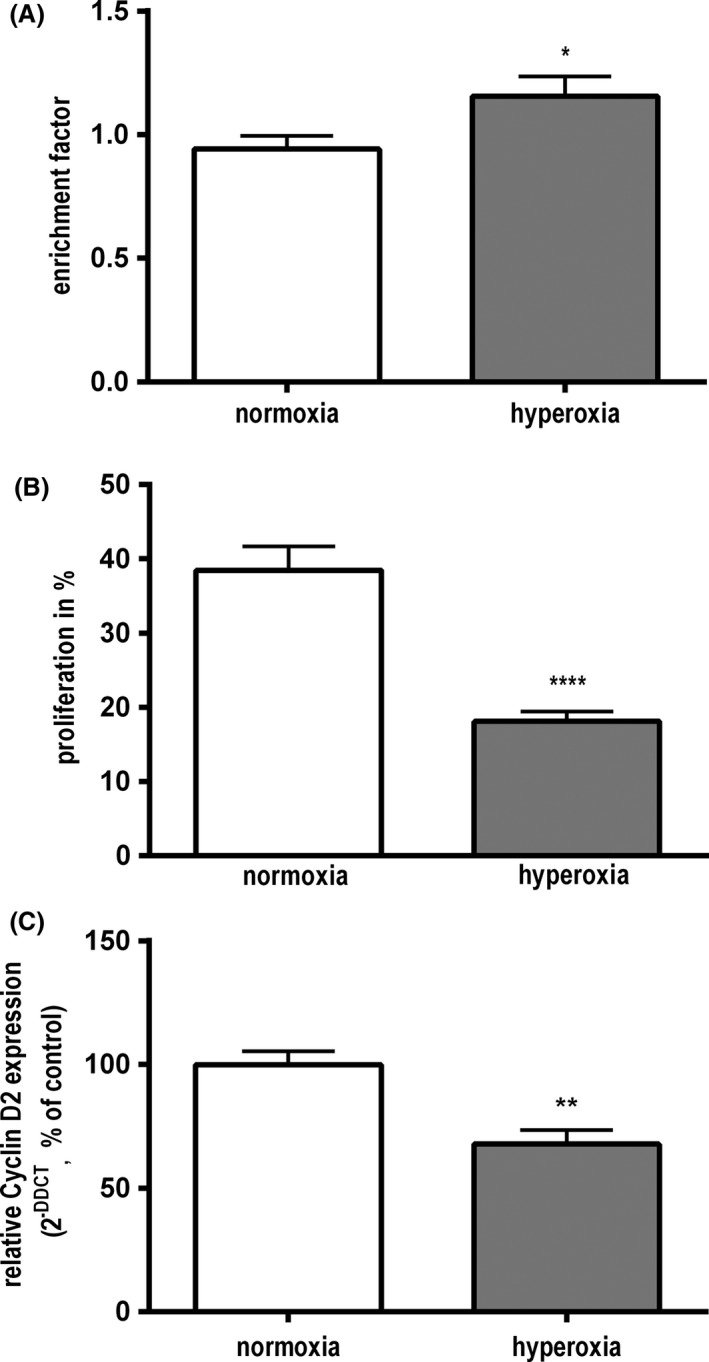
(A) Cell Death Detection ELISA ^plus^ after 24 h in hyperoxia. C8‐D1A‐astrocytes presented significant increase in mono‐ and oligonucleosomes released into cytoplasm. (data are means ± SEM;* n* = 3; students *t*‐test, *P < 0.05). (B) Flow cytometry with CFSE‐labeling after 24 h in hyperoxia. Treated astrocytes showed a significantly reduced proliferation rate in comparison to control cells in normoxia. (data are mean ± SEM;* n* = 4; students *t*‐test, ****P < 0.0001). (C) Real‐time PCR analysis after 24 h in hyperoxia. Astrocytes significantly reduced the expression of Cyclin D2. (data are mean ± SEM;* n* = 5; students *t*‐test, **P < 0.01).

Cell Death detection ELISA^plus^ was used to investigate cell death in C8‐D1A astrocytes in hyperoxia. Cell Death Detection ELISA revealed a significant rise of released mono‐ and oligonucleosomes in the cytoplasm of hyperoxia‐treated cells compared to cells in normoxia (1.16 vs. 0.94; *P* < 0.05) (Fig. 2A).

#### Hyperoxia causes inhibition of cell proliferation in C8‐D1A astrocytes

Flow cytometry was used to determine changes in proliferation. In comparison to cells incubated in normoxia_,_ astrocytes exposed to hyperoxia showed a significant reduction in cell division after 24 h. C8‐D1A astrocytes cultured in normoxia showed a proliferation rate of 38 ± 3.2%, whereas hyperoxia‐exposed cells presented a reduced cell division of 18.4 ± 1.4% (*P* < 0.0001) (Fig. 2B). After 48 h, the evaluation of proliferation by flow cytometry revealed a cell division rate of 73% for astrocytes incubated in normoxia, whereas exposition to hyperoxia resulted in a significantly lower proliferation rate of 27% (not shown).

#### Hyperoxia suppresses expression of Cyclin D2

To verify the results of flow cytometry, we used real‐time PCR to analyze the expression of the cell cycle regulating protein, Cyclin D2. After 24 h in hyperoxia, C8‐D1A astrocytes showed a significantly reduced expression of Cyclin D2 in real‐time PCR analysis. After hyperoxia, the expression of the protein was decreased to 67.8 ± 5.7% (*P* < 0.01) (Fig. 2C).

### Effects of progesterone in C8‐D1A astrocytes

#### Receptors PR‐AB and PR‐B are expressed in C8‐D1A mouse astrocytes

**Figure 3:**
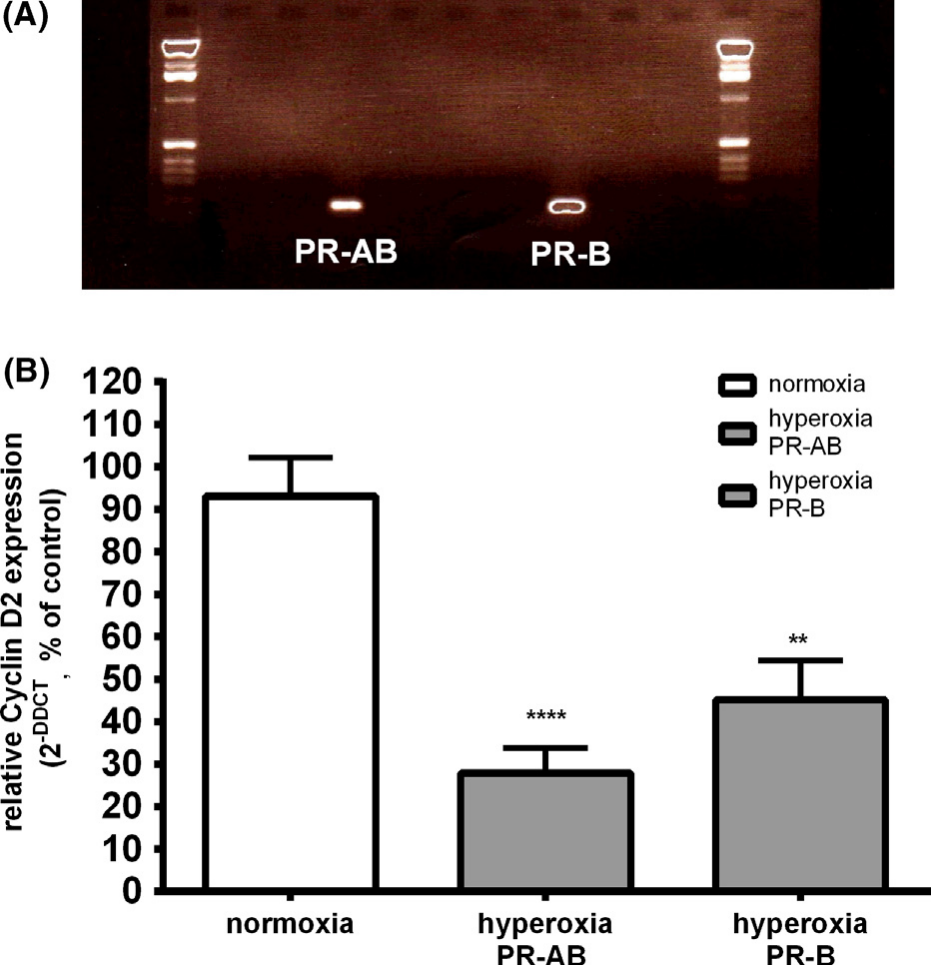
(A) Real‐time PCR analysis of progesterone receptor AB (PR‐AB) and –B (PR‐B) in normoxia. Base pair count of PR‐AB at 204 bp and PR‐B at 196 bp. Picture shows one of three experiments. (B) Real‐time PCR analysis after 24 h in hyperoxia. PR‐AB and PR‐B are significantly decreased after exposure. White column shows full expression of receptors in normoxia. (data are means ± SEM;* n* = 4; one‐way ANOVA test with Bonferroni post hoc test (**P < 0.01, ****P < 0.0001).

To determine the expression of PR‐AB and PR‐B in C8‐D1A cells, mRNA of both progesterone receptors were assessed using RT‐PCR. The investigation revealed that PR‐AB (204 bp) and PR‐B (196 bp) are both expressed in cultured cells. (Fig. 3A).

#### Impact of hyperoxia on expression of progesterone receptors

Clarifying the question why progesterone could not mediate as a protective agent against hyperoxia‐induced cell damage of astrocytes, we analyzed the quantitative expression of progesterone receptors by real‐time PCR, employing primers for PR‐AB and PR‐B receptors (see Table 1). After 24 h in hyperoxia, the expression of both receptors was significantly reduced (PR‐AB 27.9 ± 5.9%; *P* < 0.0001 and PR‐B 45.1 ± 9.3%; *P* < 0.01) in contrast to cells incubated in normoxia (Fig. 3B).

#### Progesterone does not prevent hyperoxia‐induced cell damage

**Figure 4:**
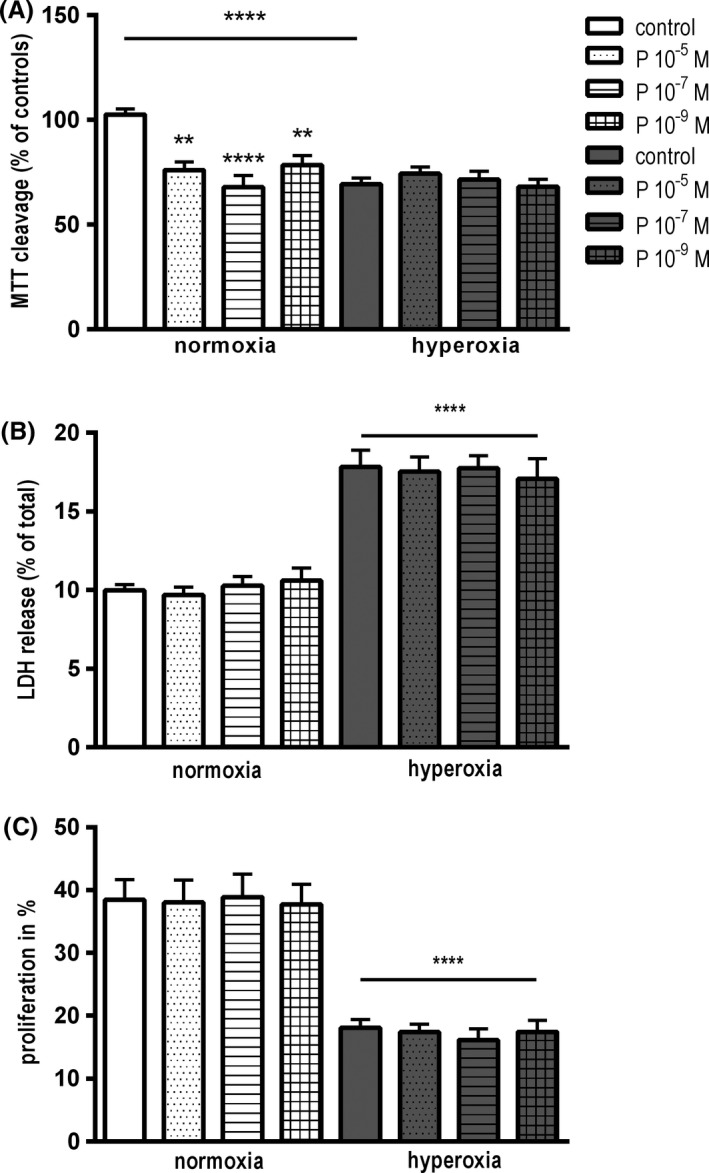
(A) Colorimetric viability assay after 24 h in hyperoxia treated with progesterone in concentrations of 10^−5^ mol/L, 10^−7^ mol/L and 10^−9^ mol/L. Cell viability of C8‐D1A astrocytes decreased significantly at presence of progesterone in normoxia and hyperoxia. Exposure to hyperoxia induced a significant reduction in cell viability exposed to untreated controls in normoxia. Progesterone at the concentrations indicated did not attenuate the hyperoxia‐induced reduction in cell viability. (data are means ± SEM;* n* = 4; one‐way ANOVA test and Bonferroni post hoc test, **P < 0.01, ****P < 0.0001). (B) Cytotoxicity detection test after 24 h in hyperoxia treated with different progesterone concentrations. Astrocytes showed significant increase in LDH release in hyperoxia. Progesterone could not decrease hyperoxia‐induced cell death. In normoxia, progesterone did not induce LDH release. (data are means ± SEM;* n* = 3; one‐way ANOVA test with Bonferroni post hoc test, ****P < 0.001). (C) Flowcytometry with CFSE‐labeling after 24 h in hyperoxia treated with different progesterone concentrations. At presence of progesterone, the proliferation rate did not change in comparison to the corresponding cells in normoxia and hyperoxia. Proliferation rate dropped under hyperoxia. (data are means ± SEM;* n* = 4; one‐way ANOVA and Bonferroni post hoc test, ****P < 0.0001).

After 24 h in normoxia, cell viability of C8‐D1A astrocytes was significantly reduced in the presence of progesterone at concentrations of 10^−5^ mol/L, 10^−7^ mol/L, and 10^−9^ mol/L (76.1 ± 3.9%; *P* < 0.01; 67.9 ± 5.5%; *P* < 0.001; 78 ± 4.6; *P* < 0.05). Moreover, the exposure of astrocytes to hyperoxia caused a significant loss of cell viability compared to control cells in normoxia (69.4 ± 2.9%; *P* < 0.001), which could not be attenuated by progesterone in all tested concentrations (74.3 ± 3.0%; *P* < 0.001; 71.6 ± 2.9%; *P* < 0.001; 68.1 ± 3.6%; *P* < 0.001) (Fig. 4A). The investigated doses of progesterone did not have an influence on LDH release in normoxia after 24 h as well as in hyperoxia. Cell death increased after 24 h of hyperoxia without any protective effect of progesterone (10.0 ± 0.4% for controls in normoxia vs. 17.8 ± 1.1% for controls in hyperoxia; *P* < 0.0001) (Fig. 4B).

#### Progesterone does not cause inhibition of cell proliferation in C8‐D1A astrocytes

To investigate the influence of progesterone on cell proliferation, flow cytometry was performed after exposure to different concentrations of progesterone. Hyperoxia for 24 h induced a significant reduction in cell proliferation compared to normoxia (38.5 ± 3.2% for controls in normoxia vs. 18.1 ± 1.4% for controls in hyperoxia; *P* < 0.0001). Progesterone showed no influence weather in hyperoxia or in normoxia (Fig. 4C).

#### Progesterone does not induce apoptosis or necrosis in C8‐D1A astrocytes

**Figure 5:**
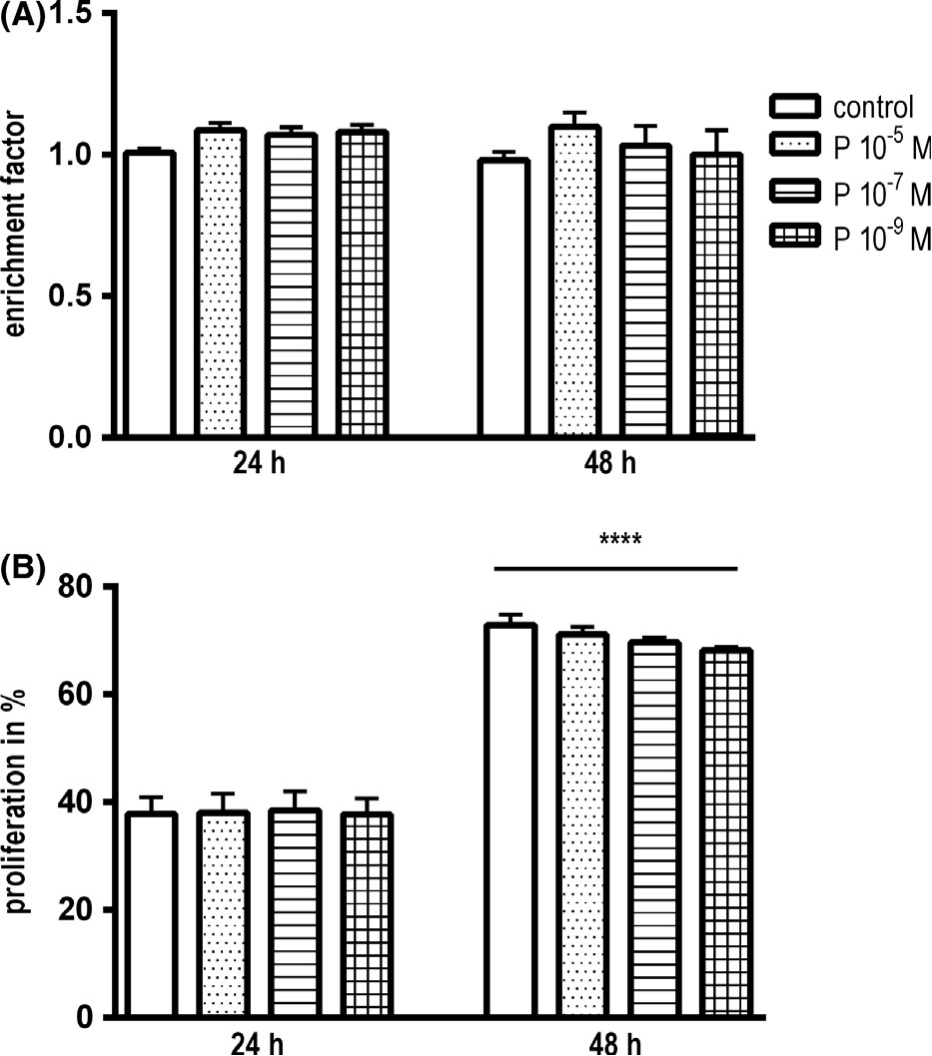
(A) Cell Death Detection ELISA ^plus^ after 24 and 48 h in normoxia treated with progesterone in concentrations of 10^−5^ mol/L, 10^−7^ mol/L, and 10^−9^ mol/L. C8‐D1A astrocytes are not affected by apoptosis at presence of progesterone. (data are means ± SEM;* n* = 3; one‐way ANOVA test and Bonferroni post hoc test) (B) Flow cytometric measurements after CFSE‐labeling after 24 and 48 h in normoxia. There is no impact of progesterone on cell proliferation within 24 h. After 48 h incubation, all cells proliferated significantly. (data are means ± SEM;* n* = 3; one‐way ANOVA test and Bonferroni post hoc test, ****P < 0.0001).

Cell Death Detection ELISA^plus^ and LDH‐assay were used to detect apoptosis and necrosis in C8‐D1A astrocytes. Figure 5A illustrates that progesterone did not induce apoptosis in C8‐D1A astrocytes during the investigated times. The enrichment factor determined by ELISA assay was not increased. Moreover, LDH‐assay revealed that progesterone did not cause elevated release of lactate dehydrogenases which excludes cell death (Fig. 4B).

#### Effects of progesterone on cell proliferation of C8‐D1A astrocytes in normoxia

Regarding the results of the colorimetric viability assay in normoxia, we wanted to define effects of progesterone on cultured astrocytes. Figure 5B shows that 24 h of incubation in normoxia did not reveal a modification of the number of proliferating cells at presence of progesterone. There was an increase in proliferation after 48 h, but no effect of progesterone (37.8 ± 3.1% to 72.9 ± 2.0%; *P* < 0.0001).

#### RU486 re‐establishes cell viability of progesterone‐treated astrocytes in normoxia

**Figure 6:**
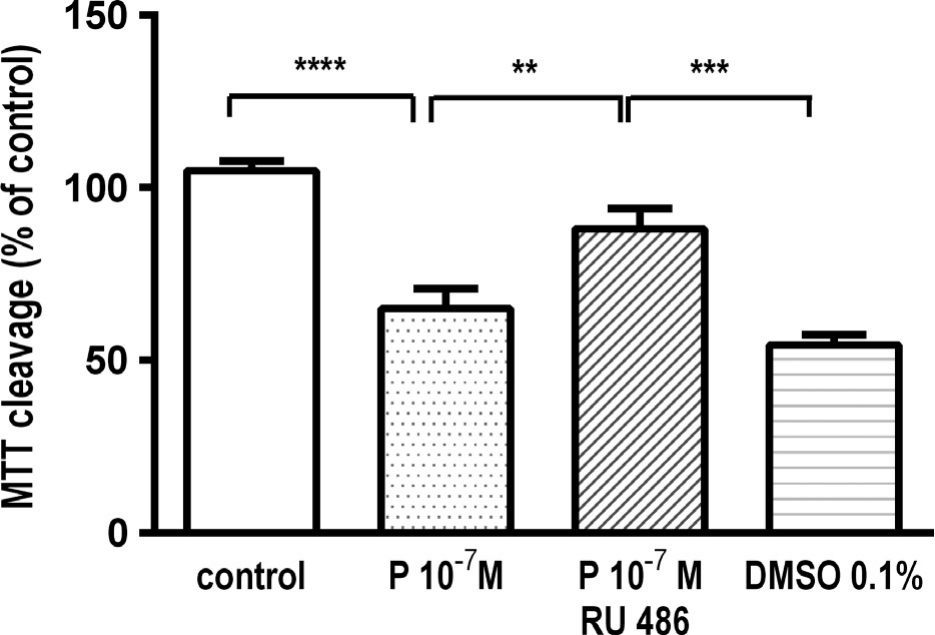
Colorimetric viability assay after 24 h in normoxia. C8‐D1A astrocytes showed a significant reduction in cell viability at presence of progesterone in a concentration of 10^−7^ mol/L. The inhibitor of the progesterone receptor RU 486 increased cell viability of progesterone‐treated cells significantly. The dissolver DMSO for RU 486 reduced cell viability. (data are means ± SEM;* n* = 5; one‐way ANOVA test and Bonferroni post hoc test, **P < 0.01, ***P < 0.001, ****P < 0.0001).

To see whether the progesterone antagonist RU486 re‐establishes the cell viability of C8‐D1A astrocytes in normoxia, we treated cells with both progesterone 10^−7^ mol/L and 10 *μ*mol/L RU486. After 24 h, astrocytes showed a significant reduction in cell viability at presence of progesterone 10^−7^ mol/L (65.0 ± 5.7%; *P* < 0.0001). RU486 antagonized this effect significantly even though the original cell amount was not achieved (88.0 ± 5.9%; *P* < 0.05). This may be explained by the DMSO effect as vehicle for RU486. DMSO alone reduced cell viability (54.4 ± 3.0%; *P* < 0.0001). The DMSO concentration was 0.1% in RU486 as well as in DMSO alone, and consequently, the effect of RU486 was reduced based on the toxicity of DMSO (Fig. 6).

## Discussion

In contrast to our initial hypothesis, experiments of this study showed that treatment with progesterone does not have a protective effect on hyperoxia‐induced cell damage of mouse C8‐D1A astrocytes. The rapid downregulation of progesterone receptors in high oxygen levels (80% O_2_) could be the key answer for this missing protection.

In our study, we described a reduced cell proliferation rate of 18 ± 1.4% due to hyperoxia. Primary astrocytes showed an inhibition of cell proliferation after 48 h exposure to 80% O_2_ measured by a significant reduction of Ki67‐labeled cells (Schmitz et al. 2011).

Moreover, progesterone showed inhibiting impacts (MTT metabolism) on cultured astrocytes in normoxia. Inhibiting effects of progesterone have been described in other experimental settings as well. In the SK‐N‐AS human neuroblastoma cell line, doses of 1 *μ*mol/L to 80 *μ*mol/L reduced significantly the reduction of cell viability in MTT‐metabolism. This effect was not blocked by RU486. Moreover, concentrations of 50 and 100 mg/kg progesterone inhibited the growth of neuroblastoma in nude mice in terms of depressing cell proliferation and inducing apoptosis (Atif et al. 2011). Exogenous administration of progesterone and its metabolite allopregnanolone may worsen hypoxic‐ischemic brain injury in immature rats. Reduced hemispheric volume and increased neuropathologic injury occurred in a dose‐ and age‐dependent fashion. This effect was especially developed in the first week of life, but emerged equally in all brain regions studied (cortex, thalamus, striatum and thalamus; Tsuji et al. 2012). Negative effects of progesterone were also seen in experimental models of stroke with ovariectomized rats. The daily supply with progesterone 1 week before an ischemic event caused exacerbated brain injury such as increased infarct size (Murphy et al. 2000). Furthermore, progesterone was shown to increase inflammation in cerebral vessels. The induction of an inflammatory response was induced by injection of the bacterial endotoxin lipopolysaccharide. Administration of estrogen significantly reduced the inflammatory response measured by cyclooxygenase 2 and nitric oxide synthase, whereas progesterone increased the inflammatory reaction (Sunday et al. 2006). Opposite effects of estrogen and progesterone also emerged when comparing the results of this study and our previous investigations, demonstrating significantly attenuated hyperoxia‐mediated apoptotic cell degeneration of C8‐D1A astrocytes following 17*β*‐estradiol administration (Huppmann et al. 2008).

According to our initial assumption, many studies describe protective qualities of progesterone in brain tissue such as reduction of infarct size (Ishrat et al. 2009) and survival rate and motor ability of mice after ischemic stroke (Gibson and Murphy 2004). Same authors reported furthermore that progesterone administration during reperfusion and before ischemia lowered brain injury (Murphy et al. 2000).

Most actions of progesterone are mediated by binding to cytoplasmic progesterone receptors or through its metabolite allopregnanolone which binds to GABA(A) receptors (Gibson et al. 2009). To what extent the C8‐D1A astrocytes are able to convert progesterone in allopregnanolone is unknown. This could explain the lack of protection in our model. Also, the metabolism could be blocked in hyperoxia. In brain cells the neuroprotective potential of progesterone may be mainly mediated by allopregnanolone via its modulation of GABA(A) receptors. Therefore, repeating the experiments in primary neuronal and astrocytic culture could confirm the thesis and should be object for further investigations.

Experiments show that the classical progesterone receptors PR play an important role in mediating neuroprotective effects of progesterone (Liu et al. 2012). However, there is evidence for membrane associated progesterone receptors mediating effects of progesterone in the CNS (Guennoun et al. 2008). So far, it is not clear if the membrane associated receptors are expressed in astrocytes and therefore could play a role in mediating neuroprotective effects in this cell type.

In conclusion, this study could not prove a protective effect of progesterone on hyperoxia‐induced cell damage in mouse astrocytes. Our experiments could not specify what part of the decrease in MTT reduction is due to inhibition of proliferation, apoptosis, or necrosis. Further investigations are required to clarify the question whether maintaining the placental hormone level after preterm birth is a promising therapeutic strategy. There is still need to specify neuroprotective elements to improve long‐time outcome of preterm infants.

## Conflict of Interest

The authors have no conflicts of interest.
